# Affordances as experienced by university faculties during and after the sudden transition to online teaching

**DOI:** 10.1016/j.heliyon.2023.e13159

**Published:** 2023-01-21

**Authors:** Najwa Alhosani, Negmeldin Alsheikh, Maxwell Peprah Opoku, Rachel Takriti, Noof M. Aljneibi, Hala Elhoweris, Rhoda Myra Garces-Bacsal

**Affiliations:** a Curriculum and Method of Instruction, United Arab Emirates University, Al-Ain, United Arab Emirates; b Special Education Department, United Arab Emirates University, Al-Ain, United Arab Emirates; c Emirates Centre for Happiness Research, UAE University, Al-Ain, United Arab Emirates

**Keywords:** COVID-19, Well-being, Opportunities, Constraints, Faculty, Online teaching, Affordance

## Abstract

The ravaging effect of COVID-19 has been felt in all spheres of life. While countries are easing their restrictions, the remnants of COVID on education remain, with most universities formally embracing online teaching. Faculty have had to deal with this sudden and enduring transition to online teaching. Although some developments have been made with online education, enormous challenges are simultaneously reported in the literature. This mixed-method study aims to assess the essence of a faculty's bionetwork of lived experience after the sudden shift to online teaching due to the pandemic. Affordance theory was used as a theoretical lens to study the benefits, challenges, and opportunities associated with online education during and post-COVID. The study data comes from faculty members at one institution (*n* = 170) and follow-up interviews with a smaller subset of participants from the same pool (*n* = 10). Path analysis and mediation analysis revealed significant differences between the participants based on nationality and gender. While the findings supported two hypotheses, the third hypothesis was not supported. Overall, the findings showed both convergence and divergence between the qualitative and quantitative data. The study incorporates recommendations for online teaching, faculty well-being, and further research based on the results.

## 1 Introduction

The sudden transition to online learning due to COVID-19 has forced university faculty to grapple with new realities [1] as they are asked to adapt [2–4]. Lall and Singh [4] noted that “this shift in education from traditional classroom learning to computer-based learning might be one of the largest educational experiments to date” (p. 48). Blankenberger and Williams [3] stated that, consequently, when one part of a system changes in unexpected circumstances, as with COVID-19, it produces a *ripple effect*, with changes occurring until an *equilibrium* is reached. This is what the education system aims to achieve in attempting to acclimate teachers (used interchangeably with faculties) and students from all education sectors to online learning.

The devastating effect of online learning on society, including the education system, cannot be overemphasized. During the outbreak of COVID-19, online learning became the only way for faculty and instructors to connect, learn, teach, and interact with their students. However, a conducive teaching environment that encompasses technology, training, and technical support is necessary before all faculties can offer accessible and quality distance education to students [5,6]. Moreover, online teaching appears enduring, with most universities globally reforming their systems to embrace online education's “new normal” [1,7]. Thus, there is a need for both human and environmental adaptations to ensure the optimal delivery of teaching and learning services [7,8]. Besides environmental changes, faculty and instructors are among the academic practitioners facing drastic adjustments to their practices, lived experiences, and well-being in response to the ample opportunities and constraints associated with shifting from face-to-face to online teaching.

Although COVID-19 and its associated threats seem to be lessening, online learning is here to stay [8], which underscores the need for continuous dialogue on the relationship between the well-being of faculties and the opportunities and constraints they face teaching online during and after the pandemic. Specifically, there is a need for research considering the experiences of faculties, learning opportunities, and the types of support they require from universities to continue teaching online. Therefore, this study attempts to understand the correlation between social well-being, technological support, the perceived benefit of online teaching, and challenges faced by university faculties.

### 1.1 Faculty experiences during COVID-19

There needs to be more research concerning faculty experiences teaching during the outbreak of COVID-19; studies in other related domains will provide a suitable framework to situate this study. For example, factors impacting online teaching have been studied in different contexts. In one Indian study, Arora and Chauhan [9] found that staff considered data usage when communicating with students via various apps. They also sought more stable applications, which were more suitable for live-streaming classes. When considering their relationships with students, Al‐Taweel et al. [10] identified that many faculties had to grapple with remaining engaged professionally and socially with students and colleagues. When learning online, students lack non-verbal cues, such as body language and facial expressions. Arora and Chauhan [9] found that faculty could not gauge students’ body language during online interactions, negatively affecting communication lines. The authors also found that some students would log in but not actively participate in the class. Aubry et al. [11] found that faculty who had children struggled with the work-life balance due to caregiving responsibilities.

Faculty have encountered many stressors at the psychological level while teaching online. For example, Aubry et al. [11] noted that faculty shared concerns about the inability to undertake projects or lab work due to COVID-19 restrictions, which may have a long-term effect on their job roles. Al‐Taweel et al. [10] reinforced that faculty involved in research may have been particularly affected since many data collection methods were suspended due to the pandemic. Hands-on clinical courses and labs also find online learning difficult [12]. Marinoni et al. [13] and Al‐Taweel et al. [10] found that faculty had been affected by canceling conferences—a necessary form of staff development. Other challenges faculty face include the inability to complete scientific projects [13] and job security anxiety [14]. Overall, life with more integrated technology is not always straightforward.

The work-from-home situation forced staff members away from teamwork and communal spaces, isolating them from colleagues. Buckley [15] found that faculty in Canada promoted online development to allow staff to maintain professional relationships from a distance. Another idea trialed in the United Kingdom during the pandemic was coordinating a virtual “huddle” amongst faculty. This online, informal meeting encouraged socialization and positive well-being [16], with staff reporting enjoyment of these relaxed events, resulting in improved well-being, team culture, and cohesion. However, the results of other studies have revealed that many teachers experienced anxiety, depression, and stress symptoms [17–19]. Thus, Moralista and Oducado [5] call for faculty to be adequately supported when embracing new teaching methods. For example, faculty training should effectively deliver online courses [12,12,20,20].

It is clear from the literature that the COVID- 19 pandemic has added considerable stressors to the lives of faculty. Additionally, some researchers have found significant differences between study participants regarding gender, well-being, and age [[17], [18], [19],^21^]. Results from such studies revealed that female instructors and teachers experienced more stress and anxiety and less engagement during COVID-19 than their male counterparts, a challenge with which they attempted to cope using some functional strategies [[17], [18], [19],^21^,^22^]. Meanwhile, no relationship has been found between teachers’ age and their level of engagement or coping [21,22]. Despite the diversity of experiences, the association between the challenges, benefits, and opportunities associated with teaching in the era of post-COVID online education has received little attention.

### 1.2 Theoretical framework

This study explores the subjective and objective realities of the faculty and its instructors dealing with online learning. Affordance theory directly explores physical and psychical phenomena [23], explicitly outlining one's relationship with the physical environment [24]. Individuals are actors expected to work within an environment who function when they are sufficiently supported to perform a given activity [25]. The atmosphere in which they work should be friendly to provide this support. Faculty members possess characteristics that could impact the “affordance” of a given behavior. However, preconditioned possibilities in the environment will determine whether such an affordance will occur. A synergy between their personal and environmental circumstances could afford faculty the opportunity to provide effective teaching services [25].

The concept derived from affordance theory is highly applicable to understanding an online learning ecology because technology grants opportunities—with some constraints—for learning within a social sphere [26]. The present paper argues that online learning depends not solely on technology but on complex contextual factors, such as the learning environment, human behavior, and learning ecology [27]. A positive relationship between the actor and their environment would lead to an affordance. In an attempt to help faculties provide effective teaching services, both the environment and the individual are expected to provide the service and cannot be considered separately. Faculty members' volition to discharge services or use their affordance to their advantage is both dependent on and independent of the environment. This volition is conditional, as faculty may not function if they are not given a conducive affordance. Likewise, this volition is dependent because the faculty's stable mental health is necessary to provide effective teaching services.

This study postulates three layers underpinning the affordance process: cognition, recognition, and behavior [25]. First, the cognition process refers to the competencies of faculties to teach online. The faculty staff should be provided with both technical support and a conducive learning environment. In the literature, it has been noted that, during COVID, while some faculty members were supported with their online teaching [13,22], others encountered challenges [7]. Specifically, Duraku and Hoxha [7] reported significant concern regarding providing effective teaching services to students. The centrality of technology offers us an opportunity to study its availability and the assistance given to faculties in the United Arab Emirates (UAE) in the context of online education.

Second, the recognition process refers to feeling supported and understanding the perceived benefit of a given phenomenon. In this study, recognition was related to the faculty's appreciation of the benefit of online teaching. For instance, faculty members must be trained to identify the perceived benefits of online teaching. However, the literature shows lapses in online teaching services provided to faculty staff [7]. Thus, there is a need for attention to be directed toward faculty's perception of the benefits of online teaching that impact their ability to teach. Moreover, it is crucial to mention that social well-being plays a fundamental role in contemporary society. COVID affected the everyday processes of individuals coming together to work and support each other. Thus, teachers need to experience positive social well-being when they educate online, as it could fundamentally influence their actual performance. The positive interplay between cognition (technology) and the affordance process (benefits and social well-being) could hint at the technology that should be deployed for successful online teaching.

Third, the behavior could be effective when individuals encounter barriers to participating in a given phenomenon. For example, such challenges could emanate from a lack of technology, an unfriendly home environment, and difficulty working with students. Therefore, it is reasonable to hypothesize that identifying the relationship between cognition and behavior could help determine whether faculty staff can provide effective online education in the post-COVID era (Hypothesis I).

Social well-being was affected due to interruptions in human relationships during the COVID pandemic. Individuals were forced to teach from their homes, and several studies reported the psychological impact of online teaching on faculty, staff, and students [28]. Therefore, it would be easier for faculty to perform their duties in a conducive environment. On the other hand, a lack of teaching support or limited knowledge of using online platforms effectively could adversely impact their social well-being upon encountering challenges. Given this, we hypothesize that complex variables predict the social well-being of faculties (Hypothesis II). More specifically, the benefits of online teaching, technological applications, and perceived challenges could predict the social well-being of faculty in the UAE.

### 1.3 Overview of the study

The outbreak of COVID-19 affected all facets of human life globally, including the UAE. The UAE is a federation of seven sheikdoms: Abu Dhabi, Ajman, Dubai, Fujairah, Sharjah, Ras Al Khaimah, and Umm Al-Quwain. The country's main economic activities are oil exploration and the hospitality industry, which have attracted prominent expatriates to live and work in the UAE. For example, in 2016, 19% of the nearly 10 million people in the UAE were nationals [29], with the remaining ∼80% being working expatriates. The outbreak of COVID-19 had dire consequences for people in such a multicultural society. In particular, the UAE joined many other nations in announcing the closure of all educational establishments (K-12 and higher education) in March 2020, which is still partially in place. This highlights the need to explore the opportunities, constraints, and areas of improvement of teaching online in the future.

The social well-being of people in such an environment should be of prime interest to educators and policymakers. In particular, expatriates may have to deal with teaching in isolation which is at odds with the tenets of social well-being that encourage interactions between people. Thus, we hypothesized that nationality moderates the relationship between cognition, perception, and actual online teaching behavior (Hypothesis III). To support teachers in providing more practical education, attempts were made to understand the social well-being predictors of faculty members teaching in the 10.13039/100016565UAE. The following research questions guided the study:

1. What factors predict the social well-being of faculty teaching at UAE universities?
2. What background variables would significantly contribute to the affordance of faculties in the UAE?
3. What is the influence of the nationality of faculty members on affordance towards online teaching in the UAE?
4. How do faculty members perceive the opportunities and challenges associated with teaching online in the UAE?

## 2 Method

The researchers searched for deep insight into the experiences of faculties and adopted a mixed method design for this study. A convergent mixed-method approach enables researchers to consolidate and validate quantitative and qualitative findings [30]. The data integration in this study involved merging the results from two datasets: faculty's perspectives gathered via an online survey and a semi-structured interview. The convergent data enriched the study by collecting large datasets, which provided a broad understanding of faculties' affordances. In addition, the follow-up study helped to explain key trends identified in the initial phase. Thus, the results reported here help broaden our understanding of faculty issues and challenges associated with distant teaching.

### 2.1 Dataset I

**Study participants**: The participants in this study were faculty from one of the premier universities in the UAE. The university has a student body of approximately 14,000, most of whom are UAE nationals, who make up 81% of the total student population. In contrast, among the approximately 1000 faculty members, 83% are expatriates. The inclusion criteria for this study were as follows: a) current faculty; b) teachers online; c) any cultural background; and d) above 18 years old and possess the capacity to consent to participate in this study.

One hundred and seventy (*n* = 170) faculty members completed the online survey. Regarding gender, 69% were male, and 31% were female. Also, 80% were expatriates, while 20% were locals (see Table 1 for details).

**Table 1:** Summary of demographic characteristics of participants.

Category (n = 170)	Sample	%
***Gender*** Female Male	53 117	31 69
***Nationality*** Emiratis Arab-speaking expat Non-Arab-speaking expat	34 61 75	20 36 44
***Age*** 30–40 y 41 years and above	30 140	18 82
***Teaching experience*** 0–5 y 6–10 y 11–15 y 16 or more years	25 28 22 95	15 16 13 56
***College*** Humanities Science	88 82	52 48
***Hours of teaching*** 1–6 h Seven or more hours	84 86	49 51

**Instrument:** A two-part instrument was used for data collection from the teachers and students. Participants’ demographic data were collected (participant type, gender, age, teaching area, nationality, and hours teaching online daily) for the first phase.

The second section revolved around the social well-being of the distance education scale (SWDES) developed for this study. The 23 items were developed from a literature review. Four sub-scales (perceived benefits of online learning [n = 6], technological application [n = 6], social well-being [n = 6], and perceived challenges [n = 5]) were anchored on a five-point Likert scale ranging from strongly disagree (1) to agree (5) (see Appendix) strongly.

**Procedure:** The study was approved by an institutional Social Sciences Ethics Committee at United Arab Emirates University. An initial email explaining the study and its protocol was sent to all faculty; then, the online survey, made using Google Forms, was distributed via email. An introduction to the survey was provided, and all participants were asked to provide their consent to participate. The data was collected between May 2020 and February 2021.

**Data analysis:** The data were transferred to Microsoft Excel for cleaning before being transferred to Statistical Package for Social Sciences for further analysis. The data were then transferred to AMOS for further analysis. Since the questionnaire was developed specifically for this study, we decided to perform confirmatory factor analysis (CFA) to assess its appropriateness based on the following indicators: chi-square degrees of freedom ratio, comparative fit index (CFI), Tucker Lewis Index (TLI), the root square error of approximation (RMSEA), standard root means square residual (SRMR), and factor loadings of at least 0.5 for each item. The cut-offs were as follows: a chi-square of less than 0.5 [31], CFI and TLI values of 0.90 or greater, RMSEA values less than 0.08, and RMSR values between 0.03 and 0.08 [32]. The research team assumed the model would fit the data if three indicators were satisfied.

Author three proceeded to answer the research questions. First, to answer research question 1, path analysis was conducted to determine which of the predictors (perceived benefit of online teaching/learning, application of technology, and perceived barriers) would influence the social well-being of the teachers and students. Next, author three checked the model's validity using the abovementioned indicators.

To answer research question 2, AMOS was used to perform mediation analysis. The independent variables (perceived benefit of online teaching/learning, application of technology, and perceived barriers) were the predictors of social well-being. In contrast, the demographic variables of interest (nationality, participation type, and gender) were used as mediators. Once again, the model's fitness was assessed using the indicators above.

### 2.2 Dataset II

A subset of 10 faculty from the original sample (*n* = 170) was selected for the interviews. The interviews had a qualitative semi-structured design and aimed to glean the faculty's experiences with the transition to online teaching. Upon completing the study's first phase, participants were asked if they wished to participate in an interview. The maximum variation technique was used to select the sample based on gender, specialization, rank, years of experience, and age (see Table 2 below). The participants who indicated a desire to participate were screened and invited to participate in the second phase. After ten interviews, the data reached saturation, thus, the recruitment discontinuation [33].

**Table 2:** Summary of interviewees (*n* = 10).

Code	Gender	College	Rank	Experience	Age
Faculty #1	Male	Engineering	Professor	17	56
Faculty #2	Male	Humanities & Social Studies	Associate Professor	14	48
Faculty #3	Female	Medicine & Health Science	Associate Professor	10	46
Faculty #4	Male	Information Technology	Associate Professor	10	52
Faculty #5	Female	Science	Assistant Professor	12	52
Faculty #6	Female	Education	Assistant Professor	10	42
Faculty #7	Male	Law	Associate Professor	16	50
Faculty #8	Male	Agriculture & Vet. Medicine	Associate Professor	12	53
Faculty #9	Male	Business & Economics	Professor	20	58
Faculty #10	Male	Humanity & Soc. Studies	Professor	18	56

The qualitative data were subjected to thematic analysis [34–36], employing the three steps suggested by Miles et al. [35]: 1) data condensation, 2) data display, and 3) drawing and verifying conclusions. In the first step, the researchers condensed the interview data by selecting, simplifying, abstracting, and transforming it. This process included reading for familiarization with the data, initial coding, and searching for themes as outlined by Braun and Clarke [34] and Nowell et al. [36]. The researchers displayed the data in the second step by organizing the interview scripts to develop categories. This step encompasses categorizing codes and tabulating them under the identified themes [34,36]. The third step enabled us to draw and verify the conclusion by “noting the patterns, explanations, and casual flows and propositions” (pp. 12–13) [35]. The following quotes were extracted from the data, with commentaries run in the data. All were shared and approved by all the co-authors.

## 3 Results

### 3.1 Dataset I

CFA was conducted to assess the validity of the 23-item scale (see Table 3). In Model 1, the fit indices met the required thresholds: chi-square = 2.63, CFI = 0.83, TLI = 0.81, RMSEA = 0.10, and SRMR = 0.08. However, on one of the sub-scales, one item had a factor loading below 0.50; thus, it was removed for another computation. In Model 2, the fit indices (chi-square = 0.267; CFI = 0.84; TLI = 0.82; RMSEA = 0.10 and SRMR = 0.07) met closely, the required thresholds, and all item loadings were above 0.50.

**Table 3:** Summary of fit-indices.

	df	χ2	CFI	TLI	RMSEA	SRMR
Model 1	224	2.63	.83	.81	.10	.08
Model 2	203	2.67	.84	.82	.10	.07
Model 3	63	3.80	.85	.81	.13	.07
Model 4	63	2.55	.92	.90	.10	.05
Model 5	42	1.75	.95	.94	.07	.06

CFI = comparative fit index; TLI = Tucker Lewis Index; RMSEA = root square error of approximation; SRMR = standard root means square residual.

**Fig. 1:**
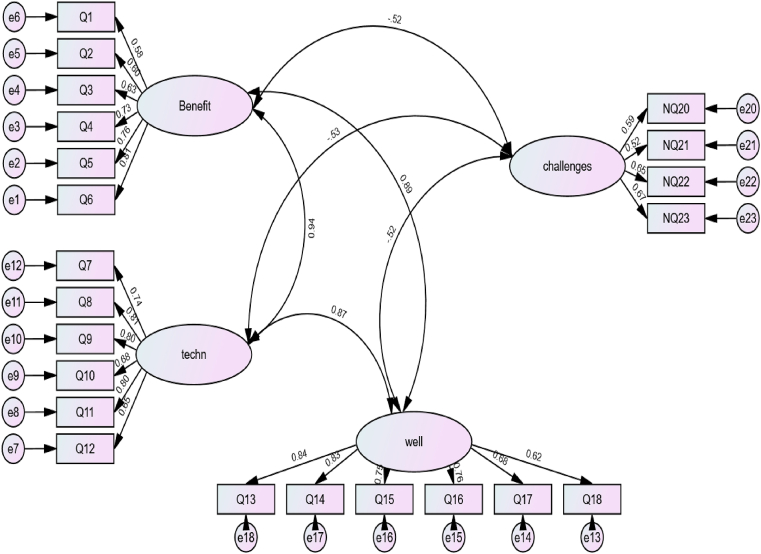
Summary of confirmatory factor analysis Note: techn = technology application; Benefit = Perceived benefit; well = social well-being; challenges = perceived challenges.

The correlations between the measures were appropriate: perceived benefit and technology, r = 0.94; technology and social well-being, r = 0.87; perceived benefit and social well-being = 0.89; social well-being and challenges = −0.52; perceived benefit and challenges = −0.52; and technology and perceived challenges = −0.53 (see Fig. 1).

Overall, 22 items were included in the analysis: perceived benefits (6 items), technological applications (6 items), social well-being (6 items), and perceived challenges (4 items). As a result, the reliability of the scale, computed using Cronbach's alpha, was as follows: perceived benefits = .84, technological application = .88, social well-being = 0.89, and perceived challenges = .70.

**Fig. 2:**
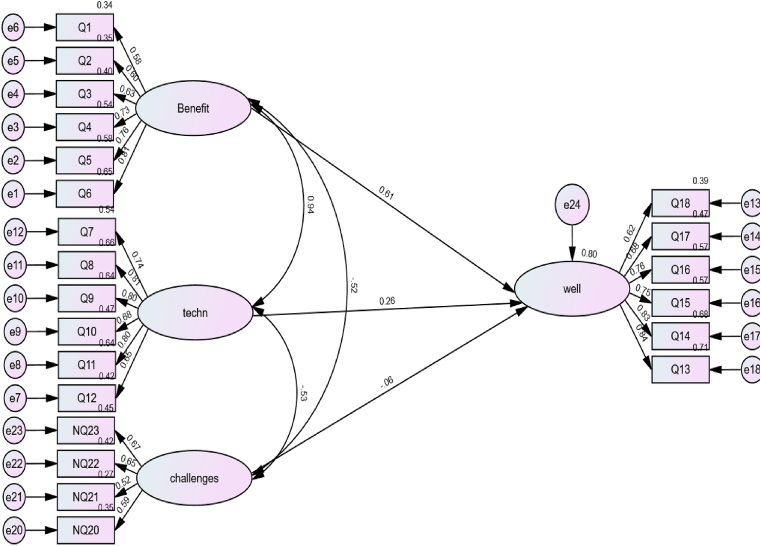
Summary of path analysis Note: techn = technology application; Benefit = Perceived benefit; well = social well-being; challenges = perceived challenges.

A path analysis was conducted to understand the other measures' ability to predict faculty's social well-being when teaching online during and after the pandemic (see Fig. 2). The model was found appropriate (the indices were the same as the imputations in Model 2). However, only perceived benefits predicted the social well-being of the participants (b = 0.62, p = .03).

T-tests and analysis of variance were conducted to understand the differences between the participants in terms of the demographic variables (see Table 4). Differences were found between the participants based on age and all other measures. While younger faculty scored high on perceived benefits (t (168) = 2.60, p = .006, Cohen's d = 0.52), technological application (t (168) = 2.60, p = .005, Cohen's d = 0.52), and social well-being (t (169) = 2.62, p = .005, Cohen's d = 0.53), older faculty members reported more challenges (t (168) = −3.11, p = .001, Cohen's d = 0.63).

**Table 4:** Demographic profile of participants and measures.

	Benefit	Technology	Well-Being	Challenges
***Gender*** Female Male *t* Cohen's d	3.48 (.90) 3.39 (.82) 0.65 0.11	3.64 (1.02) 3.49 (.85) 1.00 0.17	3.45 (1.01) 3.30 (.97) 0.80 0.15	3.75 (.84) 3.88 (.65) −0.99# 0.18
***Nationality*** Emiratis Arab-speaking expat Non-Arab-speaking expat *F* Partial eta squared	3.37 (.89) 3.43 (.84) 3.43 (84) 0.06 0.001	3.49 (1.03) 3.63 (.84) 3.48 (.90) 0.50 0.006	3.41 (.95) 3.30 (1.00) 3.36 (.99) 0.13 0.002	3.85 (.69) 3.71 (.72) 3.94 (.72) 1.71 0.02
***Age*** 30–40 y 41 years and above *t* Cohen's d	3.77 (.72) 3.34 (.85) 2.57** 0.52	3.92 (.88) 3.46 (.89) 2.60** 0.52	3.26 (.90) 3.26 (.97) 2.62** 0.53	3.48 (.77) 3.92 (.68) −3.11** 0.63
***Teaching experience*** 0–5 y 6–10 y 11–15 y 16 or more years *F* Partial eta squared	3.73 (.80) 3.39 (.70) 3.57 (.84) 3.31 (.88) 1.95 0.03	4.01 (.78)^a^ 3.33 (.84)^b^ 3.89 (.57)^a,b,c^ 3.39 (.96)^b,c,d^ 5.08** 0.08	3.73 (.75) 3.24 (.93) 3.33 (1.06) 3.29 (1.02) 1.50 0.03	3.74 (.70) 3.76 (.84) 3.65 (.69) 3.93 (.68) 1.35 0.02
***College*** Humanities Science *t* Cohen's d	3.49 (.93) 3.34 (.74) 1.13# 0.17	3.54 (1.00) 3.54 (.81) 0.04# 0.006	3.40 (1.06) 3.30 (.90) 0.68 0.10	3.80 (.80) 3.88 (.62) −0.77# 0.12
***Hours of teaching*** 1–6 h 7 or more hours *t* Cohen's d	3.40 (.88) 3.44 (.85) −0.30 0.05	3.57 (.88) 3.51 (.94) 0.47 0.07	3.36 (1.00) 3.34 (.97) 0.19 0.03	3.76 (.67) 3.92 (.76) −1.41 0.22

*p < .05; **p < .01; *Note: technology = technology application; Benefit = Perceived benefit; well = social well-being; challenges = perceived challenges*.

In terms of teaching experience, differences were found between the participants in terms of technology (F (3, 166) = 5.08, partial eta squared = 0.08). Specifically, a Post-hoc comparison using the Tukey HSD test showed that those with 0–5 years of teaching experience differed in terms of technology from those with between 6 and 10 years and those with 16 or more years.

### 3.2 Mediation analysis

Mediation analysis was done to understand the influence of nationality on the relationship between the independent variables (perceived benefits, technological application, and perceived challenges) and the dependent variable (social well-being). The models were judged to be a good fit, with the indices meeting the thresholds (see Table 2, Fit Models 3–5). Nationality was not found to influence the relationship between the independent and dependent variables. However, it is essential to state that the independent variable directly influenced the dependent variable (see Table 5).

**Table 5:** Nationality as a mediator between independent and dependent variables.

	Beta	S. E.	C. R.	P-value
Nationality Perceived benefit Nationality * perceived benefit	−0.02 0.89 0.03	0.05 0.08 0.06	−.43 7.62 .38	.67 .001** .71
Nationality Technological application Nationality * technology application	0.01 0.87 −0.005	0.05 0.11 0.21	.20 6.77 .04	.84 .001** .66
Nationality Perceived challenge Nationality * perceived challenge	0.05 −0.52 0.08	0.07 0.10 0.09	.66 −4.38 .91	.51 .001** .36

*p < .05; **p < .01.

### 3.3 Dataset II: qualitative findings

**Fig. 3:**
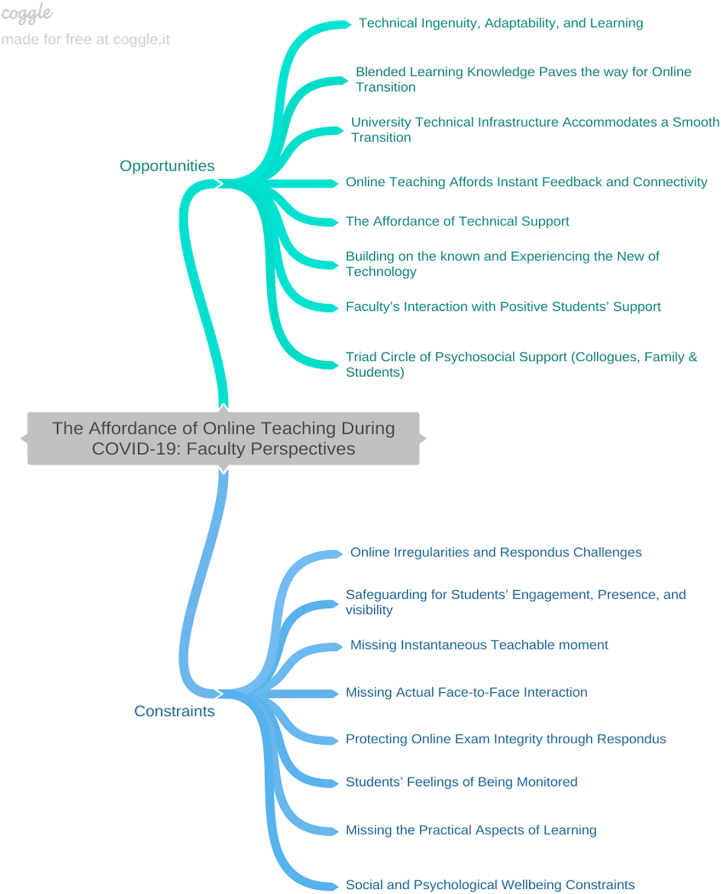
Major themes extracted from the faculty interviews.

In the interviews, the participants shared their perspectives on the influence of perceived benefits on social well-being. Participants also shared their views on the opportunities and challenges involved in online teaching. The results are grouped under two broad themes: benefits and social well-being and constraints of online teaching (see Fig. 3).

## 4 Benefits and social well-being

Participants shared their perspectives on what has enabled them to transition to distant teaching effectively. In addition, they indicated reasons which might allow them to teach effectively online due to their well-being being safeguarded. Notably, participants disagreed on the influence of nationality or age on their affordance to teach online.

### 4.1 Facilities and experience

According to the participants, the university's technological infrastructure offered the faculty and instructors viable solutions to cope with this sudden shift in teaching style. Although faculty and instructors typically use the technology provided by the university, the change to online learning compelled them to learn new techniques for interacting with students to compensate for the lack of a face-to-face experience. In addition, the sudden shift to online learning forced the faculty and instructors to learn and adapt to new technological methods quickly. Another factor that helped the faculty transfer smoothly to online learning was transferring some of their courses to blended learning (face-to-face and online teaching)— before the sudden shift to online-only learning. Faculty 1 expressed the following in this regard:

I think I have learned that, you know, as an academic, as a human being in general, we can adapt quickly. Yes, we resist change; we resist change, for example, if you have asked all the academics. When I signed up for blended learning then, I was reluctant. I signed [up] last year. (Faculty #1)

This cannot be; some stuff I wanted to do. However, I thought, “no, I do not have the time, I do not have”; I have to put in more effort. I have done it, like recording my lectures. I was doing my exams online, trying to do my quizzes and midterm online, and then I said, “oh, maybe next semester,” but now I get it because COVID forced us to do it. So, I have learned that I can do things. Plus, I feel like now I am more, I will be more giant, whatever will happen. I know I can adapt, and I can do things. (Faculty #4)

The faculties considered that the university's technological infrastructure helped them connect through online teaching and increasing learning possibilities. Most faculty members viewed this as a great opportunity that supported a smooth transition. For example, Faculty #6, who was from the physics department and had experienced different university systems, felt that the university was well equipped in terms of technological infrastructure compared to other universities in various countries around the world. Another participant said:

And again, as I have told you, we were fortunate at UAEU as students and staff to have all of these platforms already. For example, I usually do my quizzes online. So even my students were already used to doing quizzes online. Of course, it was done in class. So, I was there; it is a… it is a different experience for them. Absolutely. However, we were fortunate to have the technology overall. (Faculty #7)

#### 4.1.1 Positive faculty interactions

Most of the interviewed faculty reiterated that the technological infrastructure enabled them to interact with their students. According to the faculty, some of the affordances of online interaction during COVID-19 included the availability of course materials and the ability of students to use and reuse such resources conveniently. For example, faculty #10 mentioned the following:

So, for me, if you ask me, um, since I, uh, I enjoyed it, um, because for multiple reasons, because once, um, I can get ahold of all the resources, um, and communicated to the students, um, as an… as much as I want. And then, of course, that material is always available to the students, even if they are not at that moment in front of… um… in the class. So, they can always review it, uh, since the videos were recorded; the slides or the contents of the course were so structured, uh, since they were feeding two online classes. (Faculty #10)

During the pandemic, faculties had to stay home and work online during and after COVID-19. Most of the faculty indicated they found support from family, colleagues, and students. Despite being challenging, the faculty believed it was a time of solidarity and reassurance for these groups.

So, I think they were pretty happy. Uh, I tried my best, but this is, on a personal level, like with my students. I tried my best every single time to tell them that these were not normal conditions because you are worried. We were concerned about your family. Some of my students have, uh, children. So, you know, they have to be focused, but they must teach their children simultaneously. So, I made sure to tell them that because sometimes, as teachers, we forget also. I mean, the students forget that we are human beings with emotions and vice versa. (Faculty #6)

I live far away from my family, so our relationship is still the same. With colleagues, it did not change as well aaaaaa we did not stop contact. We kept making a couple of weekly meetings; we were in continuous contact. (Faculty #10)

I think, you know, to some extent, you can keep relationships going by meeting students online and speaking with colleagues through email or resuming online discussions. Still, it is different from being able to see them in person. (Faculty #3)

#### 4.1.2 Adaptability

Participants disagreed that younger faculty would have more technical experience than their older counterparts. For instance, they agreed that the more experienced faculty members who had years of online teaching would be able to utilize it to teach. However, many believed that the level of adaptability to online teaching could enhance teaching effectiveness. Although faculty and instructors typically used the technology provided by the university, the change to online learning compelled them to learn new techniques of interacting with students to compensate for the lack of face-to-face experiences. In addition, the sudden shift to online learning forced the faculty and instructors to adapt to and quickly learn new technologies. Another factor that helped the faculty smoothly transition to online learning was their experience transferring some of their courses to blended learning before the sudden shift to online-only classes.

I think I have learned that, you know, as an academic and as a human being in general, we can adapt quickly. Yes, we resist change; we resist change, for example, if you have asked all the academics. Now, even when I signed up for blended learning, I was reluctant to sign up last year. (Faculty #3).

So, it was very challenging. It was something to do, and we successfully did it. Moreover, I think all the students and the instructors are very proud of this, but it was very challenging. But I guess the challenge came from moving immediately into something new, and we were unprepared for that. (Faculty #4).

#### 4.1.3 Constraints

The participants discussed their major concerns that could cancel out the gains associated with online teaching. According to participants, major concerns revolved around students’ engagement and the opportunity to do hands-on activities.

##### 4.1.3.1 Student engagement, presence, and visibility

The faculties shared that ensuring students’ engagement, presence, and visibility during online classroom sessions, as these factors make the class active and dynamic. For example, Faculty #1 declared that “the most challenging part is ensuring that the students are engaged.” Additionally, Faculty #5 mentioned the following: “Well, I think it is harder to keep an eye on all the students; students can go. When you are in the classroom, you can walk around, speak to students, can invite them to participate in the discussion.”

Most faculty believed that the lack of intuitive face-to-face interactions resulted in the loss of instantaneous and direct feedback.” This is reflected by the views of faculty #2, who said: “It is a continuous interaction process between the instructor and the students to get feedback to get them through doing the things.” Similarly, Faculty #9 added, “So you have to try to ask questions and get people responding to make sure that they are at the computer paying attention.”

The university studied herein uses the Respondus monitor, a remote proctoring viable tool for universities and higher education. Respondus allows students to take exams and other types of assessments online. Most interviewed faculty viewed the use of Respondus as challenging in terms of monitoring students and checking videos for instances of cheating. Faculty #1 and #5 particularly described this as challenging: *“*Respondus is the most challenging part actually for assessment. I think we need to revise the way we assess because this type of assessment does not assess their abilities or their attainment” (Faculty #1). Faculty #5 believed that exams are more controllable in face-to-face situations: “So, I think the potential for cheating probably increases a little bit outside of the classroom. However, as I say, it is not a serious concern because the grade distribution has been normal.”

##### 4.1.3.2 Student psychological well-being

During the pandemic, some faculty viewed online learning as challenging for students. Some students feel stressed and need an easy exam; some think they are being monitored and watched as if they are onstage. Faculty #4, #6, and #2 viewed the psychological experience online as challenging: “So, it was hard for us to, you know, how to ease the situation for them while you are giving them excellent exams that are at the level that is expected to be” (Faculty #6); “So, it is stressful, to be honest with you; if I were a student, I would be stressed as well” (Faculty #2). Faculty #4 mentioned the following:

I mean, some students tend to complain about the exams or like they prefer to have an easy exam, every student does, but yeah, I find that it is a little bit challenging, more for the students than for ourselves. And then, of course, when we started using the camera, there was a layer of complication because the students knew that there was a camera on them. So, from a psychological point of view, especially the first time I had the impression watching the video, that some of them were very stressed, even outstanding students.

#### 4.1.4 Missing the practical aspects of learning

Most faculty, especially those who teach science and technology, have internships, or teach practical aspects of learning needed to transfer practical projects or sessions of hands-on learning to an online format, felt that online learning fell short of their expectations: “We do not have practical work in our field. I can imagine in the sciences that … that would be very challenging to replicate that you cannot do lab work” (Faculty #7)*.* Two other faculties also discussed the topic:

There was no online teaching there, say or something. The students are supposed to go to different institutions in the country to do their internship for like eight weeks. But because of COVID-19, they could not be able to go anywhere. (Faculty #1)

So, with the online, I would say, um, this piece where you can do more than just teaching that is missing, of course, with online, you can do fantastic teaching, but this thing, which you have to, which as a teacher, one has to do more because, uh, here, my training in Germany was that all the students should be part of the industry. (Faculty #5).

## 5 Discussion

The world is recovering from the devastating impact of COVID-19; however, its remnants remain in the education sector. Most countries have adopted online education, where teaching is provided virtually. This study attempted to provide deeper insights into the affordance of faculties teaching at a higher education institution in the UAE. The results, showing a correlation between all the components of affordance, supported Hypothesis I. While there was a positive correlation between cognition and affordance perception, the relationship between behavior and the other two components was negative. This finding indicates that, in this post-COVID era, institutions should consider the cognitive needs, benefits, and behavior associated with online teaching. Understanding the cognitive demands and environment will enable faculty members to conduct their duties effectively. For instance, it may support their knowledgeable deployment of technology for teaching and help them feel that they are working in a supportive environment. This finding is essential given that many findings have reported barriers emanating from cognition and perception [5,19]. Therefore, these two indicators must be considered and supported before faculties can conduct their duties effectively.

The second hypothesis relates to the prediction power of cognition and actual online teaching behavior on social well-being. COVID-19 denied participants human-to-human interactions, which were significantly reduced and affected by online teaching [16,20]. However, this study found that familiarizing faculty staff with the benefits of online teaching could positively impact their social well-being. This indicates the need for institutions to invest heavily in human resources to support faculty in their online teaching. The qualitative data confirmed that university readiness in the UAE contributes largely to effective online teaching. Also, faculty members’ experiences and familiarity with systems made it easy for them to transition to distant teaching. COVID-19 seems to be enduring, and the world does not know the future. It would be helpful for institutions to continue to promote online teaching and mentally prepare faculty for future related responsibilities.

It was initially hypothesized that nationality might mediate the relationship between social well-being and other measures. It is essential to mention that UAE is a multicultural society [29], which could affect how nationality influences well-being during prolonged distance learning periods. Unfortunately, our third hypothesis needed to be supported, with nationality not controlling the relationship between cognition, behavior, or social well-being. The qualitative data supported the trend identified in the earlier quantitative phase of the study. A corpus of literature has reported on the ravaging effect on several sectors of society, including education (both teaching and learning) [16,17]. This means that to promote individuals' well-being, one's nationality should not be used as a yardstick to determine the intervention required to optimize performance. However, faculties may require similar support services before they can conduct their duties effectively. Indeed, as the results showed, attention should be given to the influence of individual variables on social well-being, such as technological abilities and understanding of the benefits of online teaching, in discussions on social well-being.

There was a divergence between the participants regarding teaching experience and technological ability. While the quantitative data showed that younger participants might have an advantage in using technology for teaching, in the second phase, participants disagreed with this concept. However, there was an acceptance that those with experience using online platforms to teach may be more competent in using technology for teaching. Overall, the outbreak of COVID-19 showed the struggles of faculties in using technology to teach online [21]. This study's findings showed that no matter the age of the faculty, all have a challenge on their hands. However, one could argue that young faculty members have the advantage of being exposed to technology in their daily lives, and possibly, so do the students. This could give younger faculty an advantage in adapting quickly to new learning platforms. Conversely, with years of experience teaching online, they can transition to online teaching, as happened during the outbreak of COVID. Thus, no matter the age, there may be opportunities and challenges in the event of online teaching. This represents an essential need for institutions to tailor professional development to suit the needs of younger and older teachers.

Aside from issues pertaining to cognition, affordance perception, and behavior, there are still challenges. The results of the first phase showed that perceived challenges correlated negatively with other measures. Specifically, as challenges increased, technological application, perceived benefits, and social well-being increased. Conversely, the reverse is true. Nevertheless, the qualitative data supported a need for more challenges regarding technology, perceived benefits, and social well-being. Given this, it would be fair to conclude that there were no challenges related to the individual and the environment within which they deliver online teaching lessons. Instead, the main challenge revolved around the learner and teaching activities. This area has not yet received much scholarly attention, particularly regarding psychological well-being and student engagement during online teaching. There is no proximal connection between the learner and the teacher, making it difficult to know whether the former is following the lesson. In the same way, the opportunity for students to practice some activities has received little attention. This potentially calls for a novel approach toward engaging students during online teaching for their participation in practical activities.

### 5.1 Study limitations

The findings of this study need to be interpreted with caution due to some limitations. First, the participants were recruited from one university, potentially limiting the generalizability of the study findings. However, the university selected for this study is a federal university that attracts students and faculty from all parts of the UAE and globally. Given this, there was heterogeneity in the participants who participated in this study. Thus, the study findings may reflect national patterns. Nevertheless, future studies could compare the affordances of faculties across borders to develop broader insight into online teaching practices. Also, future studies could compare the affordances of faculty and students regarding distance education, social well-being, and affordances. Second, the data were collected remotely, without any connection or interaction between the participants and the research team. Potential bias exists as participants may select favorable options when completing the questionnaire. However, the follow-up interviews helped develop insights into participants' perceptions. Future studies could include social desirability measures in studies of affordances to understand whether responses could be influenced by a social bias which is one's quest to be seen positively.

## 6 Conclusion and policy implications

Most countries, including the UAE, are still reeling over the impact of the COVID-19 pandemic. Several countries have adopted a cautionary approach to avert future system shutdowns. On the other hand, several countries have embraced online education as a mode of lesson delivery in the education system. While notable barriers were reported as present during the pandemic outbreak [37,38], the opportunities and challenges that need to be addressed in the future still need to be explored. The present study has attempted to fill this scholarly gap by exploring faculty affordance in online teaching. The approach was to place the individual at the center and appreciate that environmental factors might impact teaching practices. Hypotheses I and II were supported by the study findings, with a relationship identified between the measures and a perceived benefit being the only predictor of social well-being. The results have shown that continual cognitive support and improved perception of online teaching are needed to promote effective online teaching in higher education in the 10.13039/100016565UAE. Moreover, the most effective way to encourage faculty well-being is by exposing them to the benefits of online teaching. The qualitative data indicated that institutional support and adaptability are essential. However, there is a need for further discussion on how faculty could engage or enhance the participation of students during online education.

The findings of this study may have implications for policymaking to enable effective online teaching in the UAE and other similar contexts. First, continual support of faculty during online teaching may be pivotal to effective teaching. This means developing an IT support system - technological assistance could help faculty teach effectively. It also means complementing social support through family and social networks that may further help faculty staff. Universities could develop a virtual program where faculty members would come online, “touch base,” and interact or share experiences. This would help facilitate their well-being and garner social support from peers. Second, tailored professional development for young and experienced faculty members may be necessary to promote effective teaching. Faculty members of all ages could be exposed to new inventions and online teaching methods. The training could focus on student engagement and participation during online education. Other training may center on how faculty staff could provide hands-on training to students during online education.

## Author contribution statement

Najwa Alhosani: Conceived and designed the experiments; Performed the experiments; Analyzed and interpreted the data; Contributed analysis tools or data; Wrote the paper.

Negmeldin Omer Alsheikh: Conceived and designed the experiments; Performed the experiments; Analyzed and interpreted the data; Contributed analysis tools or data; Wrote the paper.

Maxwell Peprah Opoku: Conceived and designed the experiments; Performed the experiments; Analyzed and interpreted the data; Contributed analysis tools or data; Wrote the paper.

Rachel Takriti: Conceived and designed the experiments; Performed the experiments; Contributed analysis tools or data; Wrote the paper.

Noof Aljneibi: Conceived and designed the experiments; Performed the experiments; Analyzed and interpreted the data; Wrote the paper.

Hala Elhoweris: Conceived and designed the experiments; Performed the experiments; Analyzed and interpreted the data; Contributed analysis tools or data; Wrote the paper.

Rhoda Myra Garces-Bacsal: Conceived and designed the experiments; Performed the experiments; Contributed analysis tools or data; Wrote the paper.

## Funding statement

This research did not receive any specific grant from funding agencies in the public, commercial, or not-for-profit sectors.

## Data availability statement

Data will be made available on request.

## Declaration of interest’s statement

The authors declare no competing interests.
