# Supplementary material for: Affordances as experienced by university faculties during and after the sudden transition to online teaching
Source: Heliyon. 2023 Jan 21;9(2):e13159. doi: 10.1016/j.heliyon.2023.e13159 (PMC9867559; doi:10.1016/j.heliyon.2023.e13159)
Supplement: Multimedia component 1 [file mmc1.docx]

**Appendix A**

**Background Questionnaire**

1. Gender: 1) Female 2) Male
2. Nationality: 1) Emirati 2) Arab (Expat), __________ 2) International (Expat), _____________
3. Your age group: 1) 40 or Below 2) 41-45 3) 46-50

4) 51-55 5) 56 and over

1. You are Teaching: 1) Undergraduate 2) Graduate 3) Mixed
2. Years of Experience: 1) 0-5 2) 6-10 3) 11-15

4) 16-20 5) 21-25 6) Over 25 years

1. College: 1) Education 2) Humanities and Social Sciences 3) Law 4) Food and Agriculture 5) Medicine and Health Sciences

6) Business and Economics 7) Science 8) Engineering

9) Information Technology

1. How much time, on average, do you spend daily on academic internet-related activities during COVID-19?
2. 3 hours or less 2) 4 to 6 hours 3) 7 to 9 hours 4) Above 10

**Faculty staff’ Perception of Teaching Online Experience -During COVID-19 (IPTOE)**

This survey aims to explore your online teaching during COVID-19 and how you cope during this period. After reading each statement, ***circle the number*** (1, 2, 3, 4, or 5) which applies to you**.**  Note that there are **no right or wrong responses** to any of the items on this questionnaire.

‘1’ means “Strongly Disagree.” ‘2’ means “Disagree**.”** ‘3’ means that “Neutral” is About **50%.**

‘4’ means “Agree.” ‘5’ means “Strongly Agree.”

| **Category** | | **Item Type** |  | | | | |  | | |  | | |  | | | |  | | | | |  |  |
| --- | --- | --- | --- | --- | --- | --- | --- | --- | --- | --- | --- | --- | --- | --- | --- | --- | --- | --- | --- | --- | --- | --- | --- | --- |
| 1. **Pedagogy -During COVID-19; Online learning enables me to: SD D N A SA** | | | | | | | | | | | | | | | | | | | | | | | |  |
| L1 | Save my time to work on research and services duties | | | 1 | | | | | | 2 | 3 | | | 4 | | | | 5 | | |  |  |  |  |
| L2 | Try new instructional tools to teach my students | | | 1 | | | | | | 2 | 3 | | | 4 | | | | 5 | | |  |  |  |  |
| L3 | Try new teaching strategies to make online learning active | | | 1 | | | | | | 2 | 3 | | | 4 | | | | 5 | | |  |  |  |  |
| L4 | Communicate with shy and reserved students and challenge them | | | 1 | | | | | | 2 | 3 | | | 4 | | | | 5 | | |  |  |  |  |
| L5 | Value the exchange of information with my students | | | | | 1 | 2 | | | | | 3 | 4 | | | | 5 | | |  |  |  |  |  |
| L6 | Become more active in teaching and pay attention to details | | | | | 1 | 2 | | | | | 3 | 4 | | | | 5 | | |  |  |  |  |  |
| 1. Technology-During COVID-19; Online learning enables me to: | | | | | | | | | | | | | | | | | | | | | | | |  |
| T1 | Use technology more effectively to fulfill my teaching objectives | | | | 1 | | | | | 2 | | 3 | | | | 4 | | 5 | | | |  |  |  |
| T2 | Connect more with my students and understand their needs | | | | 1 | | | | | 2 | | 3 | | | | 4 | | 5 | | | |  |  |  |
| T3 | Give tangible written and oral feedback to my students | | | | 1 | | | | | 2 | | 3 | | | | 4 | | 5 | | | |  |  |  |
| T4 | Use different sources of information (e.g., audio, videos, etc.) | | | | 1 | | | | | 2 | | 3 | | | | 4 | | 5 | | | |  |  |  |
| T5 | Mediate my teaching ideas, concepts and information online | | | | 1 | | | | | 2 | | 3 | | | | 4 | | 5 | | | |  |  |  |
| T6 | Relocate and distribute my time to work on other duties | | | | 1 | | | | | 2 | | 3 | | | | 4 | | 5 | | | |  |  |  |
| 1. Social & Wellbeing-During COVID-19; Online learning enables me to: | | | | | | | | | | | | | | | | | | | | | | | |  |
| S1 | Enjoy my time by connecting virtually with my students and colleagues | | | 1 | | | | | 2 | | | 3 | | | 4 | | | | 5 | | | | | |
| S2 | Work at my pace and give me a space to voice my opinions | | | 1 | | | | | 2 | | | 3 | | | 4 | | | | 5 | | | | | |
| S3 | Connect to my social and academic milieu while at home | | | 1 | | | | | 2 | | | 3 | | | 4 | | | | 5 | | | | | |
| S4 | Fulfill my needs by integrating family and work needs | | | 1 | | | | | 2 | | | 3 | | | 4 | | | | 5 | | | | | |
| S5 | Feel integrated and less socially isolated during COVID-19 | | | 1 | | | | | 2 | | | 3 | | | 4 | | | | 5 | | | | | |
| S6 | Value the sense of belonging to my institute, colleagues, and students | | | **1** | | | | | **2** | | | **3** | | | **4** | | | | **5** | | | | | |

| 1. **Challenges of Online learning: I can say ……..** |
| --- |

| C1 | My academic load increased during COVID-19 | **1** | **2** | **3** | **4** | **5** |
| --- | --- | --- | --- | --- | --- | --- |
| C2 | My teaching became more challenging during COVID-19 | **1** | **2** | **3** | **4** | **5** |
| C3 | I face some technical problems in the online learning | **1** | **2** | **3** | **4** | **5** |
| C4 | I missed the actual classroom interaction mode of learning | **1** | **2** | **3** | **4** | **5** |
| C5 | It is challenging to conduct exams online | **1** | **2** | **3** | **4** | **5** |

**Appendix B**

**Interview Questions for students**

1. Can you share with us your online learning experience during COVID-19? What do you learn from these experiences? What opportunities does this experience offer?
2. How do you benefit from the technology during COVID-19? How does online learning make you feel? Connected? Isolated? What are the challenges that you face in using technology? What are the opportunities that technology offers during this period?
3. How does this period change your ways of looking at your learning?
4. What type of support did you require during this period?
5. How does this period affect your relationship, negatively or positively? With your colleagues? Your family? Your instructors? Etc.?
6. How optimistic are you in looking at the future?
